# Boosting working memory in the elderly: driving prefrontal theta–gamma coupling via repeated neuromodulation

**DOI:** 10.1007/s11357-024-01272-3

**Published:** 2024-07-12

**Authors:** Lukas Diedrich, Hannah I. Kolhoff, Clara Bergmann, Mathias Bähr, Andrea Antal

**Affiliations:** Department of Neurology, University Medical Center Göttingen, Göttingen, Germany

**Keywords:** Working memory (WM), Transcranial alternating current stimulation (tACS), Cross-frequency coupling, Cognition, Aging, Elderly

## Abstract

The escalating global burden of age-related neurodegenerative diseases and associated healthcare costs necessitates innovative interventions to stabilize or enhance cognitive functions. Deficits in working memory (WM) are linked to alterations in prefrontal theta–gamma cross-frequency coupling. Low-intensity transcranial alternating current stimulation (tACS) has emerged as a non-invasive, low-cost approach capable of modulating ongoing oscillations in targeted brain areas through entrainment. This study investigates the impact of multi-session peak-coupled theta–gamma cross-frequency tACS administered to the dorsolateral prefrontal cortex (DLPFC) on WM performance in older adults. In a randomized, sham-controlled, triple-blinded design, 77 participants underwent 16 stimulation sessions over six weeks while performing *n*-back tasks. Signal detection measures revealed increased 2-back sensitivity and robust modulations of response bias, indicating improved WM and decision-making adaptations, respectively. No effects were observed in the 1-back condition, emphasizing dependencies on cognitive load. Repeated tACS reinforces behavioral changes, indicated by increasing effect sizes. This study supports prior research correlating prefrontal theta–gamma coupling with WM processes and provides unique insights into the neurocognitive benefits of repeated tACS intervention. The well-tolerated and highly effective multi-session tACS intervention among the elderly underscores its therapeutic potential in vulnerable populations.

**Supplementary Information:**

The online version contains supplementary material available at 10.1007/s11357-024-01272-3.

## Introduction

As the world faces an ever-increasing aging population, rates of dementia diagnosis and treatment are on the rise. Together, this is leading to a rapid increase in dementia-related healthcare costs. From 2000 to 2019, the annual growth rate was 4.5%. Estimations resulted in $263 billion in global healthcare expenditures linked to dementia in the year 2019, with a projected accumulation of $1.6 trillion by the year 2050 [1]. One strategy with which to reduce the prevalence of dementia and its associated healthcare costs is to develop effective interventions to slow down or even prevent the transition from mild cognitive impairment (MCI)—considered a preliminary stage of dementia—to full-fledged dementia. In addition to ongoing research into pharmacological solutions, the exploration of low-intensity non-invasive transcranial electrical stimulation (tES) has become increasingly established and has experienced rapid growth in recent years. Two advantages of tES are the relatively low-cost application and the absence of strong adverse effects [2]. Research on tES focuses on various cognitive domains, with potential modulation of memory being a key area [3].

Working memory (WM), a cognitive system responsible for temporarily storing and manipulating information essential for complex mental tasks [4], represents a primary target for many tES interventions. Researchers aim to restore WM functionality, which is detrimentally affected by aging and neurodegenerative diseases such as MCI or Alzheimer’s disease (AD) [5]. Effects of tES on WM depend on the specific type of stimulation applied. In both major tES subtypes—transcranial direct current stimulation (tDCS) and transcranial alternating current stimulation (tACS)—a weak electrical current is externally administered to the brain through at least two electrodes affixed to the exterior of the scalp, yielding neuromodulatory effects. However, a fundamental disparity exists in their underlying working mechanisms. The application of a constant current influences cerebral excitability, consequently affecting brain activity in the region under stimulation [6]. The administration of an alternating current, on the other hand, synchronizes cortical oscillations by means of entrainment, potentially affecting oscillatory power [7]. A recent review additionally highlights the potential of tACS to influence neuronal connectivity and neural spike timing, as well as to induce synaptic plasticity [8]. Comparing both techniques in terms of their impact on WM improvement, tDCS demonstrated a small yet statistically significant positive effect on WM [9]. However, no reliable effects were found when stimulation was not repeated, whereas, for multi-session tDCS, a moderate positive effect on WM was observed [10]. In contrast, single-session tACS demonstrated moderate effects on WM performance. Interestingly, tACS interventions can result in both enhancement and deterioration, depending on the exact frequency, phase, intensity, and montage [10].

Electroencephalogram (EEG) studies have unveiled distinct oscillatory signatures within the frontal, parietal, and sensory networks during the maintenance of WM [11]. Entrainment induced by tACS allows the direct modulation of these oscillatory dynamics [12]. The most promising enhancements in WM were achieved through the application of theta- or gamma-frequency tACS targeting the prefrontal or parietal cortex. Specifically, populations characterized by diminished WM capacities, such as the elderly and cognitively impaired patients, exhibited more pronounced cognitive improvements following tACS interventions [13].

The interaction of oscillatory components leads to cross-frequency coupling, a phenomenon that plays a functional role in neural information processing and transfer [14]. In the context of WM, Lisman and Idiart established a theory that specific informational items are encoded through gamma subcycles superimposed onto a theta wave via phase-amplitude coupling (PAC) [15]. Substantiation for this theory was derived from observations in intracranial EEG recordings acquired from the hippocampus of human epilepsy patients. The findings illustrated a reduction in the modulating theta frequency as the WM load increased [16]. Another approach supporting the theta–gamma neural code theory was provided by Alekseichuk and colleagues, who introduced theta–gamma tACS [17]. This type of neurostimulation applies a complex alternating current driven by a theta fundamental wave that is superimposed by fast gamma oscillations using PAC [17]. It was investigated as to whether healthy young adults experience changes in spatial WM performance when theta–gamma tACS is applied simultaneously, targeting their dorsolateral prefrontal cortex (DLPFC). The authors were able to confirm their hypothesis by demonstrating a sensitivity increase when gamma bursts superimposed the peaks of the theta wave, whereas trough-coupled theta–gamma tACS did not lead to any performance-enhancing effects [17]. Reaction time was not significantly affected in either case. The combination of 6 Hz theta fundamental waves and 80 Hz gamma superposition provided the largest effect size. In addition, global neocortical connectivity (i.e., global phase synchronization based on the phase lag index) was found to be increased with peak-coupled theta–gamma tACS in contrast to trough-coupled theta–gamma tACS and sham stimulation [17]. These promising results led to further applications of theta–gamma tACS to modulate cognitive processes. However, in contrast to WM, performance-enhancing effects of peak-coupled theta–gamma tACS were absent when targeting declarative memory [18] and cognitive control [19]. In contrast, trough-coupled theta–gamma tACS led to decreasing performance. To the best of our knowledge, theta–gamma tACS has yet to be employed in any study involving older individuals.

In this randomized, sham-controlled, triple-blinded research study, our objective was to assess the potential of multi-session peak-coupled theta–gamma tACS in enhancing the WM performance of older adults. The study spanned 16 days, distributed over a 6-week period. Each intervention day involved participants receiving 20 min of stimulation while concurrently engaging in *n*-back training involving the 1- and 2-back conditions. We accounted for the baseline cognitive status, assessed through the Montreal Cognitive Assessment (MoCA). The study aimed to address specific research questions, namely: (1) the capacity of peak-coupled theta–gamma tACS to positively influence WM in a vulnerable target population (the elderly) and (2) the potential benefits of a multi-session intervention. Our hypotheses posited superior enhancements in WM over the intervention series in the Active tACS group compared to the sham stimulation group. Additionally, we anticipated incremental effectiveness with an increasing number of tACS sessions.

## Materials and methods

### Participants

According to the inclusion criteria, subjects were older adults aged between 55 and 85, male or female, proficient in German, neurologically and psychiatrically healthy, had normal or corrected-to-normal vision, had no serious medical conditions or addictions to alcohol, medications, or other drugs, and were not being treated with neuroleptics, benzodiazepines, and antiepileptics during participation. In line with non-invasive brain stimulation (NiBS) safety considerations, subjects with metal brain implants, pacemakers, or a history of epilepsy or stroke were excluded. All participants were naïve as to the use of tES or cognitive training and provided written informed consent before enrollment in the study.

### Study design and procedures

**Fig. 1:**
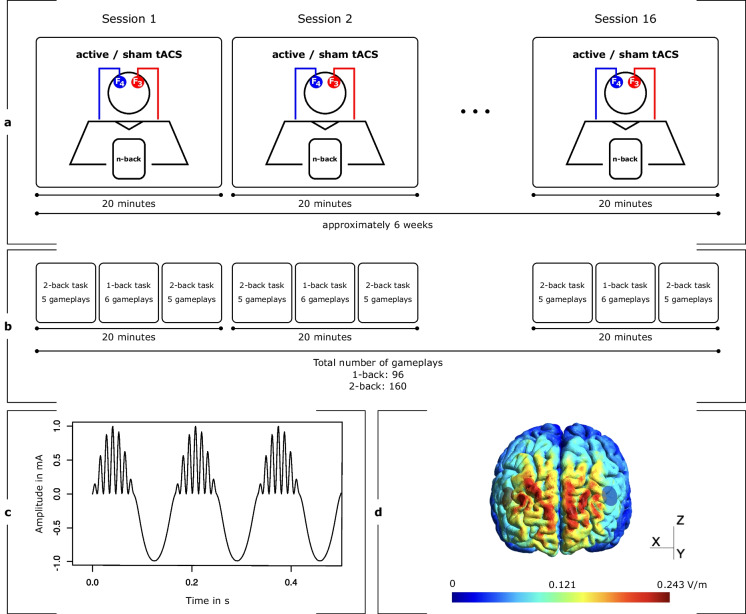
Intervention procedure. Participants underwent 16 stimulation sessions of either active or sham transcranial alternating current stimulation (tACS), targeting their dorsolateral prefrontal cortex (DLPFC) in a bilateral design. The exact electrode positions were F3 and F4, according to the international 10–20 electroencephalogram (EEG) system. Concurrent with the 20-min stimulation, subjects played two *n*-back games on a tablet (**a**). Within each session, participants initiated with five gameplays of a 2-back task, followed by six gameplays of a 1-back task and concluded with five gameplays of the 2-back task at the end of the stimulation period. In total, this equates to 96 gameplays of the 1-back task and 160 gameplays of the 2-back task (**b**). The waveform of the applied tACS paradigm with a current intensity of 2 mA peak-to-peak consists of a continuous slow frequency component of 6 Hz (theta), which is superimposed by fast gamma (80 Hz) bursts on the peaks of the theta wave by phase-amplitude coupling (**c**). A simulation of the applied electric field using SimNIBS 4 revealed a maximum field strength of 0.243 V/m over the prefrontal cortex (**d**)

The study employed a randomized, triple-blinded, sham-controlled, and parallel-group design. Prior to the initiation of the intervention series, the cognitive status of participants was assessed using the MoCA. Subsequently, subjects were exposed to 20 min of stimulation using either active or sham tACS on each of 16 intervention days. The total duration of the intervention was approximately 6 weeks. Concurrently to stimulation, cognitive *n*-back training was administered on a tablet (Fig. 1a). The *n*-back performance parameters were later analyzed to investigate the online effects (during stimulation) of tACS across multiple sessions. Ethics were approved by the Ethics Committee of the Medical Faculty of the Georg-August-University, Göttingen, Germany (7/7/2020). The principles of the Declaration of Helsinki were followed.

### N-back games

In concurrence with the application of tACS, participants played two *n*-back games using Lumosity™ (Lumos Labs, Inc.), which were executed on Samsung Galaxy Tab A tablets. While playing these virtual card games, users had to make “yes” or “no” decisions as to whether the current card stimulus matches the stimulus from *n* steps earlier. In both games, there are five distinct cards, distinguishable by either shape or color, including a green flower, a yellow triangle, a red square, a purple diamond, and a blue circle. Throughout the initial and final thirds of the stimulation duration, participants were instructed to partake in a 2-back game called memory match (MM). In the interim between these segments, they played a 1-back game referred to as speed match (SM). The duration of a single gameplay varies between the two games under examination. Specifically, a single session of SM spans a duration of 45 s, whereas MM extends for 60 s per session. Participants were instructed to engage in five consecutive sessions of MM within each of both designated segments, interspersed with six sessions of SM. This arrangement, punctuated by brief intermissions, encompassed the entirety of one stimulation session, resulting in a total duration of 20 min. After 16 interventions, participants completed 160 gameplays of MM and 96 gameplays of SM (Fig. 1b).

### Theta–gamma transcranial alternating current stimulation

Based on the findings of Alekseichuk et al., we chose their most effective tACS paradigm combining 6 Hz theta with peak-coupled 80 Hz gamma superimposition using PAC [17] (Fig. 1c). Stimulation was administered using CE-certified, battery-driven mobile stimulators (neuroConn DC-STIMULATOR MOBILE, neurocare group AG, Ilmenau, Germany). After skin disinfection, two conductive rubber electrodes (⌀ 2 cm) were attached to the participant’s scalp using conductive cream (AC Cream, Spes Medica S.r.l., Genova, Italy). Electrode placement followed a bilateral montage targeting the left and right DLPFC (F3 and F4, according to the international 10–20 EEG system). The current intensity was 2 mA (peak-to-peak). This tACS setting leads to bilateral frontal stimulation, with maximum field strengths reaching 0.243 V/m, as confirmed through electric field modeling conducted via SimNIBS 4 software, utilizing their standardized head model (Fig. 1d) [20]. Each stimulation lasted for 20 min (15 s fade in and 15 s fade out). To overcome blinding issues when stimulation is repeated [21], we modified the common sham tES protocol, which introduces an initial 30 s stimulation phase, after which the current is turned off [22]. Our modified sham tACS protocol comprised two active stimulation phases, each lasting 90 s (15 s fade in, 60 s of active stimulation at a peak-to-peak intensity of 2 mA, 15 s fade out). These active stimulation phases were administered at the initiation and termination of the 20-min intervention. Between these active stimulation blocks (i.e., in the sham phase), an 85-Hz sinusoidal current with an intensity of 50 μA peak-to-peak was applied to control impedance and ensure a stable level of impedance throughout the experiment. Stimulators were coded by a third person who was not involved in data collection or data analysis. The stimulator’s display did not convey specific details regarding the type of stimulation, ensuring blinding of experimenters and subjects.

### Statistical analyses

To examine the effects of stimulation between intervention groups on *n*-back performance, we conducted two distinct analyses using R [23] and the RStudio IDE [24]. Firstly, we treated the session (defined as an individual gameplay) as a numeric variable, ranging from 1 to 96 for SM and from 1 to 160 for MM. Secondly, we treated the stimulation session (comprising all gameplays within a given stimulation session) as a factor variable, spanning from 1 to 16 for both SM and MM. This allowed us to (1) explore the overall *n*-back performance trend throughout the intervention and (2) determine the specific number of stimulation sessions required before discernible between-group effects emerged.

In order to enhance the data quality and thus minimize potential sources of bias in the subsequent statistical analyses, several data filtering criteria were applied. Specifically, data pertaining to subjects who discontinued participation in the study were omitted (two subjects). Additionally, data associated with the initial round of each game, which were influenced by practice effects, were excluded. Furthermore, to ensure uniformity and comparability of data across participants, data derived from rounds of gameplay that exceeded predefined thresholds (SM > 96 gameplays; MM > 160 gameplays; see Fig. 1b) were also excluded from the dataset (SM: 5 gameplays; MM: 13 gameplays). These unintended gameplays were inadvertently initiated by some subjects in some sessions, contrary to the instructions given. Further gameplays were excluded if the subject gave no response (SM: 0 gameplays; MM: 8 gameplays). Lastly, in the SM dataset, data from a single participant were entirely removed due to the consistent response of “yes.”

In the analysis of the 2-back task (MM), we did not exclude the first two trials of each gameplay, as the game was designed in such a way that the user receives the initial two stimuli without the ability to respond. Consequently, the first trial corresponds to the third stimulus, yielding informative data for analysis.

To assess the *n*-back task performance, we examined three parameters from the preprocessed dataset: the sensitivity index *d* prime (*d*’), the response bias C, and the response time (RT). According to the signal detection theory, *d*’ quantifies accuracy independent of response bias, while C characterizes the participant’s tendency to make either “yes” or “no” responses [25]. Subsequent to the general data filtering described above, outlier removal was performed separately for both the signal detection measures (*d*’, C) and the RT based on their specific characteristics and recommendations.

For the calculation of *d*’ and C, we first calculated the hit rate and false alarm rate as follows:

hit rate = true positives/(true positives + false negatives).

false alarm rate = false positives/(false positives + true negatives).

Extreme outliers were defined as data points falling above Q3 + 3 × IQR or below Q1 − 3 × IQR, where Q3 and Q1 represent the third and first quantiles, respectively, and IQR denotes the interquartile range (IQR = Q3 − Q1) [26]. In the SM dataset, a total of 119 gameplays were identified as extreme outliers based on the hit rate, while 129 gameplays were flagged as extreme outliers based on the false alarm rate. Similarly, in the MM dataset, 10 gameplays were categorized as extreme outliers for the hit rate, and 8 gameplays were classified as extreme outliers for the false alarm rate. Subsequently, all identified extreme outliers were systematically removed from the further analyses of *d*’ and C. Together with the general data filtering criteria described above, this resulted in the removal of 5.8% of the data from the SM dataset and 0.9% from the MM dataset before the analyses of *d*’ and C were carried out.

In light of the increased occurrence of hit rates of one (active tACS: SM: 72.9%, MM: 14.4%; sham tACS: SM: 72%, MM: 12.5%) and zero false alarm rates (active tACS: SM: 67.5%, MM: 14.2%; sham tACS: SM: 69.4%, MM: 17.2%) in both groups, mean values were computed for each gameplay and each group, which were further used for the calculation of *d*’ and C, according to Macmillan and Kaplan [27], as follows:

$$d{\prime}={Z}_{hit rate}-{Z}_{false alarm rate}$$

$$C=-0.5\times ({Z}_{hit rate}+{Z}_{false alarm rate})$$

where *Z* corresponds to the *z*-score.

In order to ensure reliability when performing RT analysis, we excluded gameplays where responses were faster or slower than the average response time ± 3 × standard deviation (SM: 78 gameplays; MM: 139 gameplays), as this method was shown to introduce only a small bias, while not excluding outliers was associated with the largest absolute bias when analyzing RTs [28]. Together with the general data filtering criteria described above, this resulted in the removal of 3.5% of the data from the SM dataset and 1.9% from the MM dataset before RT analysis was performed.

The changes in *d*’ and C throughout stimulation sessions and comparisons between intervention groups were further examined using linear models (LMs). To investigate changes in RT, we applied generalized linear models (GLMs) because of the positively skewed gamma-like distribution of RTs and the biases that would be associated with transforming the data [29]. Due to the logarithmic relationship between the number of gameplays and RT, we applied the logarithmic link function to our gamma regression models. To assess the potential impact of incorporating a second-order polynomial for the variable “Gameplay” while controlling for variations in age and baseline MoCA scores, we integrated these variables as covariates within our statistical models. Both age and MoCA values were mean-centered. We evaluated their significant impact by comparing models with and without these variables and applying the likelihood ratio test (using the *model.comparison* function in R, which is integrated into the *flexplot* package [30]). In LMs and GLMs, the likelihoods are represented by the Akaike information criteria (AIC), with a lower AIC indicating greater information gain [31]. The full documentation on the model results and model optimizations can be found in Supplementary information S1. All LMs and GLMs employed met the requisite assumptions, including linearity, absence of collinearity, normality, and homoscedasticity, the final two of which apply solely to LMs. Influential data points were detected and removed as described above. Post hoc analyses were conducted using the emmeans package (Lenth, 2021). Effect sizes were computed utilizing Cohen’s d (Cohen, 1988) and categorized as very small (|d|< 0.2), small (0.2 <|d|< 0.5), moderate (0.5 <|d|< 0.8), and large (|d|> 0.8). Employing the *report* package, we included key model metrics, such as *R*^2^, adjusted *R*^2^, beta coefficients, 95% confidence intervals (CI), *t*-values, *p*-values, standardized beta coefficients (std. beta), and standardized confidence intervals (std. CI), as advocated by Makowski et al. [32]. Figures were generated employing the *ggplot2* and *sjplot* packages [33, 34].

## Results

### Baseline characteristics and group differences

**Fig. 2:**
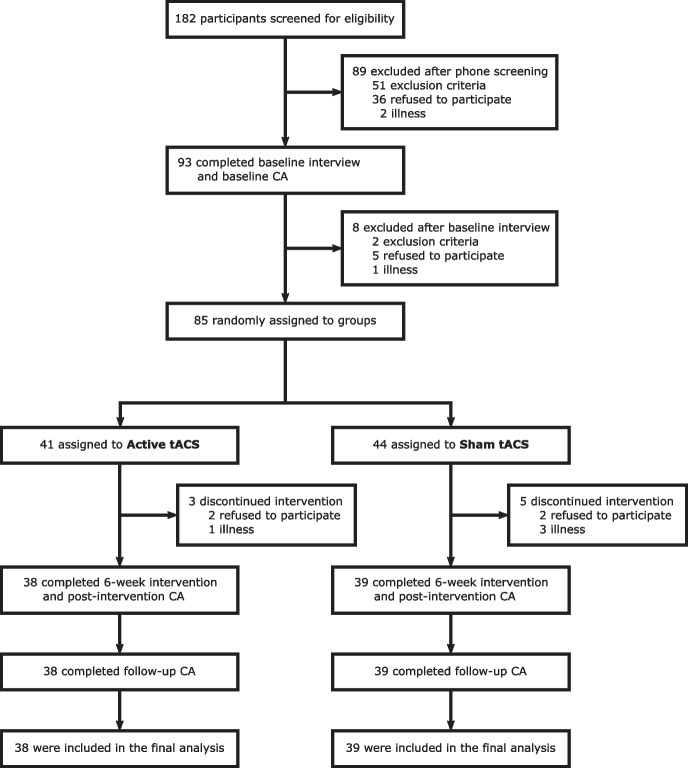
Participant flow diagram. CA = cognitive assessment; tACS = transcranial alternating current stimulation

A total of 182 participants were screened for eligibility. Of these, 53 were identified as ineligible due to the presence of one or more exclusion criteria, 41 declined to participate, and three experienced health-related issues, resulting in a final enrollment of 85 participants who were randomly allocated (1:1) to either active tACS or sham tACS. Subsequent to the baseline interview, three subjects from each experimental group withdrew for unspecified reasons or due to health concerns. During the intervention period, two additional subjects from the sham tACS group discontinued their participation, citing personal reasons. Ultimately, a total of 77 participants (45 female, age range: 55–84 years, mean age: 69.8 ± 7.17 years), comprising 38 in the active tACS group and 39 in the sham tACS group, successfully completed the 16-day interventional series (Fig. 2). Groups were equal in terms of age, gender distribution, education, and baseline cognition (Table 1).

**Table 1:** Baseline characteristics

	Active tACS (*n* = 38)	Sham tACS (*n* = 39)	*p*-value
Age, years	69.4 (7.6)	70.3 (6.8)	0.59
Gender (F/M)	21/17	24/15	0.74
Education, years	13.4 (5.2)	13.3 (3.5)	0.89
MoCA baseline	25.4 (3.1)	25.5 (3)	0.89

The data comprises descriptive statistics, presenting mean values and standard deviations (in parentheses) for age (measured in years), years of education, and baseline Montreal cognitive assessment (MoCA) scores. Gender distribution is indicated by case numbers designating female (F) and male (M) subjects. The column labeled *p*-value shows the statistical disparities observed between the intervention groups at baseline. Two-sample, two-sided *t*-tests were employed to assess the variables of age, education, and MoCA scores, while the chi-squared (χ^2^) test was utilized to compare gender distribution across intervention groups. A *p*-value of ≤ 0.05 denoted statistical significance

*tACS*, transcranial alternating current stimulation

### No tACS-induced modulations of 1-back performance

**Fig. 3:**
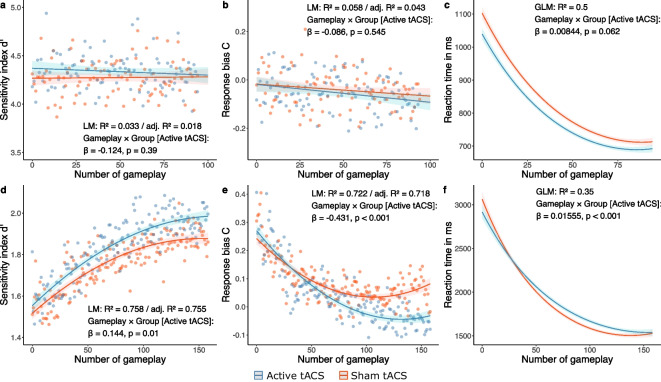
Predicted progression of 1-back and 2-back task performance across sequential gameplays. Linear models (LMs) were fitted to predict the progression of the sensitivity index *d*’ and the response bias C for both the 1-back (**a**–**b**) and the 2-back (**d**–**e**) task across sequential gameplays comparing the Active tACS (blue) with the Sham tACS (red) intervention group. Final LMs for the 1-back task included Gameplay (1–96) and Group (Active tACS vs. Sham tACS) as predictors (formula: *d*’ / C ~ Gameplay × Group). Final LMs for the 2-back task included Gameplay^2^ as an additional predictor (formula: *d*’ / C ~ Gameplay × Group + Gameplay^2^). The scatter plot illustrates the participant group mean values for each gameplay, distinguishing between the Active tACS group (blue dots) and the Sham tACS group (red dots). Generalized linear models (GLMs) were fitted to predict the reaction time for both the 1-back (**c**) and 2-back (**f**) tasks. Final GLMs included Gameplay (for 1-back: 1–96; for 2-back: 1–160), Group (Active tACS vs. Sham tACS), age, and baseline Montreal cognitive assessment (MoCA) score as predictors (formula: RT ~ Gameplay × Group + Gameplay^2^ × Group + age + MoCA_baseline_). The solid lines, along with color-shaded error bands, depict regression fits and their associated 95% confidence intervals. β represents the standardized coefficient, and a *p*-value of  ≤ 0.05 indicates statistical significance for the interaction effect of Gameplay × Group [Active tACS] against reference (Gameplay × Group [Sham tACS]). Model metrics were represented by *R*^2^ and adjusted *R*^2^ for the LMs and Nagelkerke’s *R*^2^ for the GLMs. tACS = transcranial alternating current stimulation

To analyze the effect of stimulation on 1-back *d*’, we fitted an LM to predict *d*’ with Gameplay (1–96), Group (Active tACS vs. Sham tACS), age, and baseline MoCA. According to the model optimization process (Supplementary Table S1.1.1), the final best LM (formula: *d*’ ~ Gameplay × Group) explained a statistically non-significant and weak proportion of variance (*R*^2^ = 0.03, *F*(3, 186) = 2.14, *p* = 0.096, adj. *R*^2^ = 0.02). The effect of Gameplay was statistically non-significant and positive (beta = 1.45e-04, 95% CI [− 1.24e-03, 1.53e-03], *t*(186) = 0.21, *p* = 0.836; Std. beta = 0.02, 95% CI [− 0.18, 0.22]), such that *d*’ was not changing throughout the gameplays. The effect of Group [Active tACS] was statistically non-significant and positive (beta = 0.10, 95% CI [− 3.33e-03, 0.21], *t*(186) = 1.91, *p* = 0.058; Std. beta = 0.33, 95% CI [0.05, 0.62]), such that subjects from both intervention groups showed the same amount of *d*’. The effect of Gameplay × Group [Active tACS] was statistically non-significant and negative (beta =  − 8.54e-04, 95% CI [− 2.81e-03, 1.10e-03], *t*(186) =  − 0.86, *p* = 0.390; Std. beta =  − 0.12, 95% CI [− 0.41, 0.16]), such that participants from both groups performed equally over the number of gameplays (Fig. 3a).

To analyze the effect of stimulation on 1-back C, we fitted an LM to predict C with Gameplay (1–96), Group (Active tACS vs. Sham tACS), age, and baseline MoCA. According to the model optimization process (Supplementary Table S1.1.3), the final best LM (formula: C ~ Gameplay × Group) explained a statistically significant and weak proportion of variance (*R*^2^ = 0.06, *F*(3, 186) = 3.82, *p* = 0.011, adj. *R*^2^ = 0.04). The effect of Gameplay was statistically non-significant and negative (beta =  − 4.97e-04, 95% CI [− 1.05e-03, 5.55e-05], *t*(186) =  − 1.77, *p* = 0.078; Std. beta =  − 0.18, 95% CI [− 0.38, 0.02]), such that C was not changing throughout the gameplays. The effect of Group [Active tACS] was statistically non-significant and negative (beta =  − 1.54e-03, 95% CI [− 0.04, 0.04], *t*(186) =  − 0.07, p = 0.943; Std. beta =  − 0.17, 95% CI [− 0.45,0.11]), such that subjects from both intervention groups showed the same amount of C. The effect of Gameplay × Group [Active tACS] was statistically non-significant and negative (beta =  − 2.40e-04, 95% CI [− 1.02e-03, 5.41e-04], *t*(186) =  − 0.61, *p* = 0.545; Std. beta =  − 0.09, 95% CI [− 0.37, 0.19]), such that participants from both groups exhibited the same level of C over the gameplays (Fig. 3b).

To analyze the effect of stimulation on 1-back RT, we fitted a GLM to predict RT with Gameplay (1–96), Group (Active tACS vs. Sham tACS), age, and baseline MoCA. According to the model optimization process (Supplementary Table S1.1.5), the final best GLM (formula: RT ~ Gameplay × Group + Gameplay^2^ + age + MoCA_baseline_) explained a substantial proportion of variance (Nagelkerke’s *R*^2^ = 0.50). The effect of Gameplay was statistically significant and negative (beta =  − 9.93e-03, 95% CI [− 0.01, − 9.27e-03], *t*(7116) =  − 29.30, *p* < 0.001; Std. beta =  − 0.13, 95% CI [− 0.13, − 0.12]), such that RT was decreasing throughout the gameplays. The effect of Group [Active tACS] was statistically significant and negative (beta =  − 0.06, 95% CI [− 0.08, − 0.04], *t*(7116) =  − 6.53, *p* < 0.001; Std. beta =  − 0.04, 95% CI [− 0.05, − 0.04]), such that subjects from the Active tACS group had overall lower RTs. The effect of Gameplay^2^ was statistically significant and positive (beta = 5.63e-05, 95% CI [4.97e-05, 6.30e-05], *t*(7116) = 16.64, *p* < 0.001; Std. beta = 0.04, 95% CI [0.04, 0.05]), indicating a quadratic relationship between the squared value of Gameplay and mean RT. Together with the linear decline represented by the effect of Gameplay described above, a curvilinear trend emerges that represents the performance ceiling that subjects reach with an increased number of gameplays. The effect of Gameplay × Group [Active tACS] was statistically non-significant and positive (beta = 3.10e-04, 95% CI [− 1.52e-05, 6.35e-04], *t*(7116) = 1.87, *p* = 0.062; Std. beta = 8.44e-03, 95% CI [− 4.14e-04, 0.02]), such that participants from both groups performed equally over the gameplays (Fig. 3c).

Overall, repeated theta–gamma tACS showed no effect on 1-back performance. Participants in both active and sham groups exhibited similar trends in sensitivity, response bias, and RT.

### Repeated active theta–gamma tACS affects 2-back performance

To analyze the effect of stimulation on 2-back *d*’, we fitted another LM to predict *d*’ with Gameplay (1 − 160), Group (Active tACS vs. Sham tACS), age, and baseline MoCA. The final best LM (formula: *d*’ ~ Gameplay × Group + Gameplay^2^) explained a statistically significant and substantial proportion of variance (*R*^2^ = 0.76, F(4, 313) = 245.62, *p* < 0.001, adj. *R*^2^ = 0.76) (Supplementary Table S1.1.2). The effect of Gameplay was statistically significant and positive (beta = 4.74e-03, 95% CI [4.04e-03, 5.43e-03], *t*(313) = 13.35, *p* < 0.001; Std. beta = 0.73, 95% CI [0.66, 0.81]), such that subjects improved in *d*’ throughout the gameplays. The effect of Group [Active tACS] was statistically significant and positive (beta = 0.04, 95% CI [5.73e-03, 0.07], *t*(313) = 2.33, *p* = 0.020; Std. beta = 0.50, 95% CI [0.40, 0.61]), such that subjects from the Active tACS group had overall higher values of *d*’. The effect of Gameplay^2^ was statistically significant and negative (beta =  − 1.55e-05, 95% CI [− 1.97e-05, − 1.14e-05], *t*(313) =  − 7.37, *p* < 0.001; Std. beta =  − 0.23, 95% CI [− 0.29, − 0.17]), indicating a quadratic relationship between the squared value of Gameplay and mean *d*’. Together with the linear increase represented by the effect of Gameplay described above, a curvilinear trend emerges that represents the performance ceiling that subjects reach with an increased number of gameplays. The effect of Gameplay × Group [Active tACS] was statistically significant and positive (beta = 4.47e-04, 95% CI [1.07e-04, 7.88e-04], *t*(313) = 2.59, *p* = 0.010; Std. beta = 0.14, 95% CI [0.03, 0.25]), such that participants from the Active tACS group exhibited a greater improvement in *d*’ with increasing gameplays (Fig. 3d).

To analyze the effect of stimulation on 2-back C, we fitted another LM to predict C with Gameplay (1–160), Group (Active tACS vs. Sham tACS), age, and baseline MoCA. The final best LM (formula: C ~ Gameplay × Group + Gameplay^2^) explained a statistically significant and substantial proportion of variance (*R*^2^ = 0.72, *F*(4, 313) = 203.11, *p* < 0.001, adj. *R*^2^ = 0.72) (Supplementary Table S1.1.4). The effect of Gameplay was statistically significant and negative (beta =  − 3.88e-03, 95% CI [− 4.38e-03, − 3.38e-03], *t*(313) =  − 15.29, *p* < 0.001; Std. beta =  − 0.49, 95% CI [− 0.57, − 0.41]), such that subjects’ C decreased across gameplays. The effect of Group [Active tACS] was statistically significant and positive (beta = 0.03, 95% CI [5.71e-03, 0.05], *t*(313) = 2.47, *p* = 0.014; Std. beta =  − 0.45, 95% CI [− 0.56, − 0.33]), such that subjects from the Active tACS group had overall higher values of C. The effect of Gameplay^2^ was statistically significant and positive (beta = 1.81e-05, 95% CI [1.52e-05, 2.11e-05], *t*(313) = 12.02, *p* < 0.001; Std. beta = 0.40, 95% CI [0.34, 0.47]), indicating a quadratic relationship between the squared value of Gameplay and mean C. Together with the linear decline represented by the effect of Gameplay described above, a curvilinear trend emerges that represents the performance ceiling that subjects reach with an increased number of gameplays. The effect of Gameplay × Group [Active tACS] was statistically significant and negative (beta =  − 8.94e-04, 95% CI [− 1.14e-03, − 6.51e-04], *t*(313) =  − 7.22, *p* < 0.001; Std. beta =  − 0.43, 95% CI [− 0.55, − 0.31]), such that participants from the Active tACS group exhibited a greater decline in C with increasing gameplays (Fig. 3e).

To analyze the effect of stimulation on 2-back RT, we fitted a GLM to predict RT with Gameplay (1–160), Group (Active tACS vs. Sham tACS), age, and baseline MoCA. The final best GLM (formula: RT ~ Gameplay × Group + Gameplay^2^ × Group + age + MoCA_baseline_) explained a substantial proportion of variance (Nagelkerke’s *R*^2^ = 0.35) (Supplementary Table S1.1.6). The effect of Gameplay was statistically significant and negative (beta =  − 0.01, 95% CI [− 0.01, − 9.62e-03], *t*(12,073) =  − 27.81, *p* < 0.001; Std. beta =  − 0.20, 95% CI [− 0.21, − 0.19]), such that RT was decreasing throughout the gameplays. The effect of Group [Active tACS] was statistically significant and negative (beta =  − 0.05, 95% CI [− 0.08, − 0.01], *t*(12,073) =  − 2.69, *p* = 0.007; Std. beta = 0.05, 95% CI [0.03, 0.07]), such that subjects from the Active tACS group had overall lower RTs. The effect of Gameplay^2^ was statistically significant and positive (beta = 3.76e-05, 95% CI [3.31e-05, 4.20e-05], *t*(12,073) = 16.55, *p* < 0.001; Std. beta = 0.08, 95% CI [0.07, 0.09]), which represents the performance ceiling, as described above. The effect of Gameplay × Group [Active tACS] was statistically significant and positive (beta = 2.09e-03, 95% CI [1.06e-03, 3.12e-03], *t*(12,073) = 3.94, *p* < 0.001; Std. beta = 0.02, 95% CI [3.71e-03, 0.03]), such that participants from the Sham tACS group exhibited a faster reduction in RT with increasing gameplays. The effect of Gameplay^2^ × Group [Active tACS] was statistically significant and negative (beta =  − 1.10e-05, 95% CI [− 1.73e-05, − 4.66e-06], *t*(12073) =  − 3.39, *p* < 0.001; Std. beta =  − 0.02, 95% CI [− 0.04, − 9.68e-03]), indicating that subjects belonging to the Active tACS group displayed a slightly attenuated positive quadratic relationship between the squared value of Gameplay and mean RT (Fig. 3f).

In summary, repeated peak-coupled theta–gamma tACS selectively influences 2-back performance, with increased sensitivity and notable modulation of response bias observed in the Active stimulation group compared to sham. Conversely, the Active tACS group showed a slight attenuation in the reduction of RT across gameplays.

### Enhanced effect sizes with increasing number of stimulations on 2-back sensitivity and response bias

To examine and quantify between-group effects of stimulation at different stimulation sessions, we first fitted LMs to predict 2-back *d*’ and 2-back C with Stimulation Session (1–16), Group (Active tACS vs. Sham tACS), age, and baseline MoCA. Similarly, a GLM was fitted to predict 2-back RT. Subsequently, we calculated estimated marginal means (EMMs) and performed pairwise comparisons with the Bonferroni correction method. Lastly, we quantified the practical significance of these differences by calculating Cohen’s d effect sizes.

**Fig. 4:**
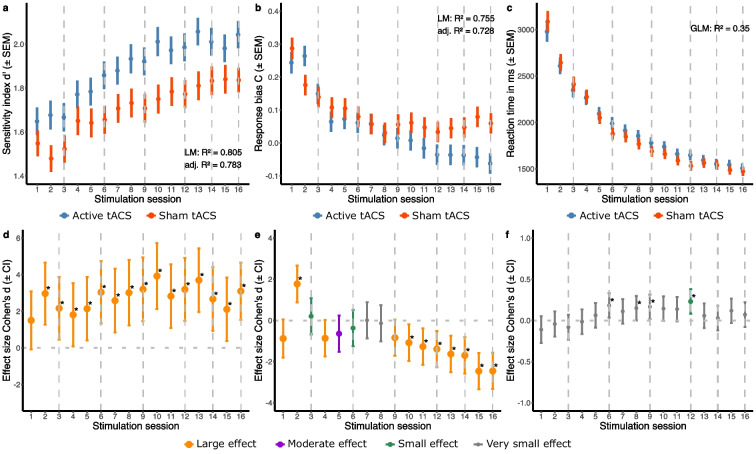
Predicted progression of 2-back task performance and between-group effect sizes across sequential stimulation sessions. Linear models (LMs) were fitted to predict the progression of the sensitivity index *d*’ and the response bias C for the 2-back task (**a**–**b**) across sequential stimulation sessions comparing the Active tACS (blue) with the Sham tACS (red) intervention group. The final LM for *d*’ included Stimulation Session (1–16), Group (Active tACS vs. Sham tACS), and age as predictors (formula: *d*’ ~ Stimulation Session × Group + age). The final LM predicting C included Stimulation Session and Group as predictors (formula: C ~ Stimulation Session × Group). A generalized linear model (GLM) was fitted to predict 2-back reaction time (**c**) with Stimulation Session (1–16), Group (Active tACS vs. Sham tACS), age, and baseline Montreal cognitive assessment (MoCA) score (formula: RT ~ Stimulation Session × Group + age + MoCA_baseline_). Dot-whisker plots depict the mean ± standard error of the mean (SEM). Model metrics were represented by *R*^2^ and adjusted *R*^2^ for the LMs and Nagelkerke’s *R*^2^ for the GLM. Cohen’s d effect sizes ± 95% confidence intervals were computed for between-group effects at each stimulation session, for *d*’ (**d**), C (**e**), and reaction time (**f**). Effect sizes were classified as very small (|d|< 0.2), small (0.2 <|d|< 0.5), moderate (0.5 <|d|< 0.8), and large (|d|> 0.8). A *p*-value of ≤ 0.05* indicates statistical significance between groups. tACS = transcranial alternating current stimulation

To predict 2-back *d*’ at stimulation sessions, the optimized LM (formula: *d*’ ~ Stimulation Session × Group + age) explained a statistically significant and substantial proportion of variance (*R*^2^ = 0.81, *F*(32, 285) = 36.83, *p* < 0.001, adj. *R*^2^ = 0.78) (Supplementary Table S1.2.1). Post hoc pairwise comparisons revealed statistically significant differences between intervention groups at all stimulation sessions except for the first session (EMMeans_Diff_ = 0.1, 95% CI [− 0.01, 0.21], p_bonf_ = 0.06). The corresponding Cohen’s d effect sizes were large (|d|> 0.8) and showed an almost linear increasing stimulation effect starting from session 3 (EMMeans_Diff_ = 0.14, 95% CI [0.03, 0.26], *p*_bonf_ = 0.01, Cohen’s d = 2.2) and reaching a maximum effect size at session 10 (EMMeans_Diff_ = 0.26, 95% CI [0.14, 0.38], *p*_bonf_ < 0.001, Cohen’s d = 3.92). Between stimulation sessions 11 and 16, there was no clear trend of increasing or decreasing effect sizes (Fig. 4a,d) (see Supplementary Table S1.2.2 for full post hoc results).

To predict 2-back C at stimulation sessions, the optimized LM (formula: C ~ Stimulation Session × Group) explained a statistically significant and substantial proportion of variance (*R*^2^ = 0.75, *F*(31, 286) = 28.40, *p* < 0.001, adj. *R*^2^ = 0.73) (Supplementary Table S1.2.3). Post hoc testing demonstrated statistically significant differences between intervention groups at session 2 and from session 10 to session 16. The corresponding Cohen’s d effect sizes were large (|d|> 0.8) and showed an almost linear decreasing stimulation effect starting from session 7 (EMMeans_Diff_ = 0.00, 95% CI [− 0.04, 0.04], *p*_bonf_ = 0.98, Cohen’s d = 0.01) and reaching a maximum effect size at session 15 (EMMeans_Diff_ =  − 0.12, 95% CI [− 0.17, − 0.08], *p*_bonf_ < 0.001, Cohen’s d =  − 2.46). Between the first and seventh stimulation sessions, there was no clear trend of increasing or decreasing effect sizes (Fig. 4b,e) (see Supplementary Table S1.2.4 for full post hoc results).

To predict 2-back RT at stimulation sessions, the optimized GLM (formula: RT ~ Stimulation Session × Group + age + MoCA_baseline_) explained a substantial proportion of variance (Nagelkerke’s *R*^2^ = 0.35) (Supplementary Table S1.2.5). Post hoc pairwise comparisons indicated statistically significant differences between intervention groups at stimulation sessions 6, 8, 9, and 12, such that subjects from the Sham tACS group had faster RTs. The corresponding Cohen’s d effect sizes were very small (|d|< 0.2) for sessions 6, 8, and 9, and small (|d|< 0.5) for session 12 (EMMeans_Diff_ = 0.07, 95% CI [0.03, 0.12], *p*_bonf_ = 0.002, Cohen’s d = 0.23) and showed no positive or negative trend in terms of size (Fig. 4c,f) (see Supplementary Table S1.2.6 for full post hoc results).

### A 16-day treatment-like tACS intervention is well-tolerated by elderly people

We confirm the tolerability of administering multi-session tACS to elderly people, as evidenced by the absence of moderate adverse effects (AEs), serious adverse effects (SAEs), or side effects (SEs) in both the Active and Sham tACS groups. Additionally, participants in both groups reported comparable incidences of mild AEs and SEs, primarily characterized by tingling sensations (62% in the active group; 65% in the sham group). Other AEs or SEs, such as skin irritations, headaches, nervousness, fatigue, vertigo, or phosphenes, occurred in 2% of cases or less (refer to Supplementary Table S2 for specific quantities and group differentials). Furthermore, effective blinding was demonstrated, as 57% of participants correctly guessed the applied stimulation type post-intervention, as indicated by Pearson’s chi-squared test (*χ*^2^ = 2.29, *p* = 0.32).

## Discussion

Our study provides evidence for targeted improvement of WM in older adults via repeated non-invasive neuromodulation using tACS. Specifically, driving prefrontal theta–gamma coupling in a treatment-like 16-day interventional series leads to enhanced sensitivity associated with a modulation of response bias. Additionally, we have shown that repeated stimulations increase the impact of intervention. Conversely, a marginal decelerating effect was noted in relation to the process of reducing RT through training. Notably, significant behavioral changes were only observed in the 2-back but not in the 1-back condition, highlighting increased tACS efficacy in cognitively demanding tasks. The results were not biased in terms of age or baseline cognitive level of participants, as these parameters were incorporated into our analyses. Our findings support the growing body of literature emphasizing the capacity of tACS to enhance cognitive functions in the elderly population [35].

The utilization of theta–gamma tACS in research is notably scarce, and a cognition-enhancing effect has so far only been reported by Alekseichuk and colleagues [17]. Our findings, demonstrating improved 2-back sensitivity through the concurrent application of peak-coupled theta–gamma tACS over the DLPFC, further substantiate the hypothesis associating theta–gamma coupling in the prefrontal area with WM [36]. Consistent with the results of Alekseichuk et al. [17], RT was not positively affected. Furthermore, the absence of tACS-induced modulatory effects in the 1-back condition reveals a dependence on cognitive load. This aligns with prior research indicating heightened efficacy of tACS in tasks characterized by greater cognitive demands [37, 38]. In contrast to our cross-frequency tACS paradigm, most other tACS studies aimed at improving WM administered either single-frequency theta or gamma tACS. Hoy et al. reported post-intervention improvements in 3-back performance among young adults following single-session gamma tACS over the DLPFC, compared with tDCS and sham stimulation [37]. However, an opposite effect was observed in schizophrenia patients [39]. In older people, a 4-day regimen of gamma high-definition tACS (HD-tACS) applied to the DLPFC exhibited a modulation of long-term memory without affecting WM. In contrast, targeting the left inferior parietal lobule with theta HD-tACS enhanced WM in an age-matched cohort [40]. The concurrent stimulation of the prefrontal cortex and the left temporal cortex using advanced multi-focal in-phase (tuned to the individual theta frequency) HD-tACS montage led to immediate and long-lasting improvements in WM of elderly participants following a single stimulation session [41]. Further investigations demonstrated increased WM capacity following individualized theta tACS over the parietal cortex, but no effect after stimulating the left DLPFC [42, 43]. Wolinski and colleagues corroborated their findings, demonstrating enhanced WM capacity following 4 Hz theta tACS over the parietal cortex [44]. In all three studies, young healthy adults were investigated. Taken together, despite some negative results (e.g., [45]), the entrainment of endogenous theta and gamma oscillations within the prefrontal or parietal cortex through diverse tACS approaches holds promise for WM modulation [10]. Our results highlight the evident potential of theta–gamma co-stimulation, emphasizing its effectiveness in elderly people and thereby contributing to limited research on this demographic.

Indeed, the majority of studies exploring potential WM modulation through tACS have been conducted in young adults, including Alekseichuk et al. [17]. However, the aging process involves structural changes in the human brain, including progressive atrophy and increased cerebrospinal fluid volume [46], thereby affecting intracerebral conductivity. In older individuals, tES simulations indicated a decline in current density with advancing age and brain atrophy [47]. This also applies to neurodegenerative patients, including those with MCI or AD, who also undergo brain shrinkage [48, 49]. Consequently, results from young healthy adults are not equally transferable to a population of older people. Nevertheless, our findings confirm prior research indicating enhanced WM performance through tACS in elderly people or those with cognitive impairments, underscoring a particular potential for these vulnerable populations [13]. Notably, Grover et al. demonstrated increased tACS efficacy for participants with lower baseline cognition, as measured by the MoCA [40]. This may be attributed to diminished WM skills observed in older adults and individuals with cognitive deficits [5]. In contrast, young healthy adults experience peak performance in WM, potentially causing ceiling effects of the intervention [50].

To the best of our knowledge, we are the first to provide evidence for a modulation of response bias (criterion) in a cognitive task through the tACS application. Comparable studies either did not explore a modulation of response criterion or could not prove it (e.g., [41]). Our findings revealed a more pronounced decrease in response criterion across successive sessions in the Active tACS group compared to sham stimulation. Consequently, repeated theta–gamma tACS targeting the DLPFC resulted in subjects answering “yes” more often [24], indicating an impact on decision-making and risk-taking processes, prompting subjects to adopt a less conservative and more liberal approach [51]. Based on two investigations, Dantas et al. reported a frequency, intensity, and hemispheric specificity of prefrontal tACS effects on risk-taking behavior. Notably, both studies emphasized the importance of frontal theta oscillations in guiding risky decision-making [52, 53].

Only a limited number of studies have employed a multi-session tACS design to elicit behavioral changes. To our knowledge, Grover et al. were the first to provide insights into repeated tACS effects targeting WM [40]. In their study, a significant boost in WM performance was only observed from the third day of the 4-day stimulation series, but persisted for 1 month post-intervention. Our findings substantiate the potential associated with the repeated application of tACS. Specifically, the between-group effect for sensitivity was increasing across sessions in favor of the active stimulation group, reaching maximum efficacy at session 10. On the other hand, multi-session tACS seems to be necessary for affecting response bias, as stable group differences only emerged after ten stimulation sessions. This could explain the lack of modulatory effects on the response criterion in Reinhart and Nguyen [41]. A recent study by Khan et al. investigated the underlying effects of multi-session tACS [54]. After 3 consecutive days of individual theta tACS targeting the left DLPFC while performing arithmetic training, participants exhibited increased resting-state functional connectivity in the frontoparietal network. However, no discernible behavioral changes or alterations in the structural integrity of the white matter were observed [54]. Overall, cumulative effects of repeated tACS promise enhanced and longer-lasting modulatory effects, but this requires further research.

Our study stands out due to a comparatively large number of subjects (*n* = 77) and the high number of repeated tACS applications (16 times). The latter, in particular, distinguishes our design from that of most other comparable studies. We thus provide insights into the tolerability of such a treatment-like intervention using tACS, which was well-tolerated by the elderly subjects in our study and did not result in moderate AEs or SAEs. However, a comprehensive understanding of the underlying effects necessitates the incorporation of neuroimaging modalities such as EEG, magnetoencephalography, or functional magnetic resonance imaging, which were absent in our study. A recent similar study by Kraft et al. demonstrated increased task-based functional connectivity in older adults following repeated combined bifrontal tDCS and CT intervention, compared to sham tDCS and CT. Additionally, their findings indicated a trending positive correlation between task performance and functional connectivity in the active tDCS group [55]. Therefore, changes in task-based functional connectivity could be a potential working mechanism for cognitive enhancements through multi-session tES and should be re-examined in future studies. Moreover, our study lacks insights into potential offline and long-term effects on WM modulation, as no *n*-back executions were conducted at later time points post-intervention.

In conclusion, we provide evidence for selective enhancement of WM processes via non-invasive tACS-induced modulation of ongoing prefrontal theta–gamma activity, confirming the pioneering results of Alekseichuk et al. [17]. The observed modulation of response bias suggests a tendency towards more liberal and less conservative cognitive patterns [51]. Such shifts could potentially foster cognitive flexibility in real-world scenarios and may facilitate social integration and interaction. Importantly, by investigating elderly people instead of young adults, we gain novel insights into the effectiveness within a population that may constitute a primary target for prospective therapeutic applications. Furthermore, we particularly highlight the tolerability and potential of multi-session tACS to maximize intervention efficacy. This could motivate future investigations into home-based tACS applications as an effective intervention method due to its low cost and high practicability, akin to previous at-home tDCS studies [56]. A comprehensive understanding of the underlying effects holds promise for further boosting the modulatory impact of tACS (e.g., through refining parameters in a personalized setting [8]). Ultimately, this progression may pave the way for the overarching goal of establishing a non-pharmacological treatment option for neurodegenerative diseases, potentially mitigating healthcare expenditures by postponing the need for care interventions.

## Supplementary Information

Below is the link to the electronic supplementary material.

Supplementary file1 (PDF 1534 KB)

## Data Availability

Data and analysis pipeline are available from the corresponding author upon reasonable request.
