# Supplementary material for: Boosting working memory in the elderly: driving prefrontal theta–gamma coupling via repeated neuromodulation
Source: GeroScience. 2024 Jul 12;47(2):1425–40. doi: 10.1007/s11357-024-01272-3 (PMC11979004; doi:10.1007/s11357-024-01272-3)
Supplement: Supplementary file 1 — Supplementary file1 (PDF 1534 KB) [file 11357_2024_1272_MOESM1_ESM.pdf]

Supplementary Materials

S1. Full Results of the Statistical Models and Model Comparisons

Tables summarizing the outcomes of linear models and generalized linear models were generated utilizing the *gtsummary* package [1]. Model comparisons were performed using the *flexplot* package [2].

S1.1 Statistical Models Predicting n-back Performance Across Gameplays and Between Groups

S1.1.1 Sensitivity Index *d'* in the 1-back Task Across Gameplays

	d' ~ gameplay × group				...+ gameplay²				...+ age				...+ MoCA_BL			
**Predictor**	**N**	**Beta**	**95% CI**	**p-value**	**N**	**Beta**	**95% CI**	**p-value**	**N**	**Beta**	**95% CI**	**p-value**	**N**	**Beta**	**95% CI**	**p-value**
gameplay	190	0.00	0.00, 0.00	0.8	190	0.00	0.00, 0.01	0.14	190	0.00	0.00, 0.00	0.8	190	0.00	0.00, 0.00	0.6
group				0.058				0.057				0.4				**0.039**
Sham tACS	95	—	—		95	—	—		95	—	—		95	—	—	
Active tACS	95	0.10	0.00, 0.21		95	0.10	0.00, 0.21		95	0.05	-0.09, 0.20		95	0.14	0.01, 0.27	
gameplay * group	190			0.4	190			0.4	190			0.5	190			0.4
gameplay * Active tACS	95	0.00	0.00, 0.00		95	0.00	0.00, 0.00		95	0.00	0.00, 0.00		95	0.00	0.00, 0.00	
I(gameplay²)					190	0.00	0.00, 0.00	0.13								
age									190	-0.06	-0.18, 0.06	0.3				
MoCA_BL													190	0.12	-0.14, 0.38	0.4
R²		0.033				0.045				0.039				0.038		
Adjusted R²		0.018				0.025				0.018				0.017		
AIC		-91.1				-91.4				-90.2				-90.0		
σ		0.187				0.187				0.187				0.188		
**Likelihood ratio test**																
**p-value**						0.133				0.313				0.359		

Table S1.1.1: Model optimization for predicting 1-back *d'* across gameplays. We fitted different linear models to predict the sensitivity index *d'* in the 1-back task with Gameplay (1–96), Group (Active tACS vs. Sham tACS), age, and baseline Montreal Cognitive Assessment (MoCA_BL) score. The table contains full results of the predictors and model metrics (R^2, adjusted R^2, AIC, sigma σ). Models were compared using the likelihood ratio test (*model.comparison* in R), assessing the significant impact of a specific term added to the model (adapted model) versus the model without this term (current best model). The p-value of the likelihood ratio test refers to the comparison between the new, adapted model with the last best model (from left to right). $p \leq 0.05$ indicates statistical significance (highlighted in bold). For the prediction of 1-back *d'*, this optimization process resulted in the following best model: *d'* ~ Gameplay × Group. tACS = transcranial alternating current stimulation; CI = confidence interval; AIC = Akaike Information Criteria.

S1.1.2 Sensitivity Index *d*’ in the 2-back Task Across Gameplays

	d' ~ gameplay × group				...+ gameplay^2				...+ gameplay^2 × group				...+ gameplay^2 + age				...+ gameplay^2 + MoCA_BL			
**Predictor**	**N**	**Beta**	**95% CI**	**p-value**	**N**	**Beta**	**95% CI**	**p-value**	**N**	**Beta**	**95% CI**	**p-value**	**N**	**Beta**	**95% CI**	**p-value**	**N**	**Beta**	**95% CI**	**p-value**
gameplay	318	0.00	0.00, 0.00	**<0.001**	318	0.00	0.00, 0.01	**<0.001**	318	0.00	0.00, 0.01	**<0.001**	318	0.00	0.00, 0.01	**<0.001**	318	0.00	0.00, 0.01	**<0.001**
group				**0.032**				**0.020**				0.6				**0.018**				0.2
Sham tACS	159	—	—		159	—	—		159	—	—		159	—	—		159	—	—	
Active tACS	159	0.04	0.00, 0.07		159	0.04	0.01, 0.07		159	0.01	-0.04, 0.06		159	0.12	0.02, 0.22		159	0.03	-0.01, 0.06	
gameplay * group	318			**0.017**	318			**0.010**	318			**0.037**	318			**0.010**	318			**0.010**
gameplay * Active tACS	159	0.00	0.00, 0.00		159	0.00	0.00, 0.00		159	0.00	0.00, 0.00		159	0.00	0.00, 0.00		159	0.00	0.00, 0.00	
I(gameplay^2)					318	0.00	0.00, 0.00	**<0.001**	318	0.00	0.00, 0.00	**<0.001**	318	0.00	0.00, 0.00	**<0.001**	318	0.00	0.00, 0.00	**<0.001**
group * I(gameplay^2)									318			0.14								
Active tACS * I(gameplay^2)									159	0.00	0.00, 0.00									
age													318	0.10	-0.01, 0.21	0.082				
MoCA_BL																	318	-0.12	-0.33, 0.10	0.3
R^2		0.716				0.758				0.760				0.761				0.759		
Adjusted R^2		0.714				0.755				0.756				0.757				0.755		
AIC		-726				-775				-775				-776				-774		
σ		0.077				0.071				0.071				0.071				0.071		
**Likelihood ratio test**																				
**p-value**						**<0.001**				0.137				0.082				0.3		

Table S1.1.2: Model optimization for predicting 2-back *d*’ across gameplays. We fitted different linear models to predict the sensitivity index *d*’ in the 2-back task with Gameplay (1-160), Group (Active tACS vs. Sham tACS), age, and baseline Montreal Cognitive Assessment (MoCA_BL) score. The table contains full results of the predictors and model metrics (R^2, adjusted R^2, AIC, sigma σ). Models were compared using the likelihood ratio test (*model.comparison* in R), assessing the significant impact of a specific term added to the model (adapted model) versus the model without this term (current best model). The p-value of the likelihood ratio test refers to the comparison between the new, adapted model with the last best model (from left to right). $p \leq 0.05$ indicates statistical significance (highlighted in bold). For the prediction of 2-back *d*’, this optimization process resulted in the following best model: *d*’ ~ Gameplay × Group + Gameplay^2. tACS = transcranial alternating current stimulation; CI = confidence interval; AIC = Akaike Information Criteria.

S1.1.3 Response Bias C in the 1-back Task Across Gameplays

	C ~ gameplay × group				...+ gameplay^2				...+ age				...+ MoCA_BL			
**Predictor**	**N**	**Beta**	**95% CI**	**p-value**	**N**	**Beta**	**95% CI**	**p-value**	**N**	**Beta**	**95% CI**	**p-value**	**N**	**Beta**	**95% CI**	**p-value**
gameplay	190	0.00	0.00, 0.00	0.078	190	0.00	0.00, 0.00	0.9	190	0.00	0.00, 0.00	0.088	190	0.00	0.00, 0.00	0.2
group				>0.9				>0.9				0.7				0.7
Sham tACS	95	—	—		95	—	—		95	—	—		95	—	—	
Active tACS	95	0.00	-0.04, 0.04		95	0.00	-0.04, 0.04		95	-0.01	-0.07, 0.05		95	0.01	-0.04, 0.07	
gameplay * group	190			0.5	190			0.5	190			0.6	190			0.5
gameplay * Active tACS	95	0.00	0.00, 0.00		95	0.00	0.00, 0.00		95	0.00	0.00, 0.00		95	0.00	0.00, 0.00	
I(gameplay^2)					190	0.00	0.00, 0.00	0.4								
age									190	-0.01	-0.06, 0.03	0.6				
MoCA_BL													190	0.04	-0.06, 0.15	0.4
R^2		0.058				0.061				0.059				0.062		
Adjusted R^2		0.043				0.041				0.039				0.041		
AIC		-440				-439				-438				-439		
σ		0.075				0.075				0.075				0.075		
**Likelihood ratio test**																
**p-value**						0.42				0.607				0.401		

Table S1.1.3: Model optimization for predicting 1-back C across gameplays. We fitted different linear models to predict the response bias C in the 1-back task with Gameplay (1–96), Group (Active tACS vs. Sham tACS), age, and baseline Montreal Cognitive Assessment (MoCA_BL) score. The table contains full results of the predictors and model metrics (R^2, adjusted R^2, AIC, sigma σ). Models were compared using the likelihood ratio test (*model.comparison* in R), assessing the significant impact of a specific term added to the model (adapted model) versus the model without this term (current best model). The p-value of the likelihood ratio test refers to the comparison between the new, adapted model with the last best model (from left to right). $p \leq 0.05$ indicates statistical significance (highlighted in bold). For the prediction of 1-back C, this optimization process resulted in the following best model: C ~ Gameplay × Group. tACS = transcranial alternating current stimulation; CI = confidence interval; AIC = Akaike Information Criteria.

S1.1.4 Response Bias C in the 2-back Task Across Gameplays

	C ~ gameplay × group				...+ gameplay²				...+ gameplay² × group				...+ gameplay² + age				...+ gameplay² + MoCA_BL			
**Predictor**	**N**	**Beta**	**95% CI**	**p-value**	**N**	**Beta**	**95% CI**	**p-value**	**N**	**Beta**	**95% CI**	**p-value**	**N**	**Beta**	**95% CI**	**p-value**	**N**	**Beta**	**95% CI**	**p-value**
gameplay	318	0.00	0.00, 0.00	**<0.001**	318	0.00	0.00, 0.00	**<0.001**	318	0.00	0.00, 0.00	**<0.001**	318	0.00	0.00, 0.00	**<0.001**	318	0.00	0.00, 0.00	**<0.001**
group				**0.041**				**0.014**				0.5				0.2				0.12
Sham tACS	159	—	—		159	—	—		159	—	—		159	—	—		159	—	—	
Active tACS	159	0.03	0.00, 0.05		159	0.03	0.01, 0.05		159	0.01	-0.02, 0.04		159	0.05	-0.02, 0.12		159	0.02	-0.01, 0.05	
gameplay * group	318			**<0.001**	318			**<0.001**	318			0.6	318			**<0.001**	318			**<0.001**
gameplay * Active tACS	159	0.00	0.00, 0.00		159	0.00	0.00, 0.00		159	0.00	0.00, 0.00		159	0.00	0.00, 0.00		159	0.00	0.00, 0.00	
I(gameplay²)					318	0.00	0.00, 0.00	**<0.001**	318	0.00	0.00, 0.00	**<0.001**	318	0.00	0.00, 0.00	**<0.001**	318	0.00	0.00, 0.00	**<0.001**
group * I(gameplay²)									318			0.2								
Active tACS * I(gameplay²)									159	0.00	0.00, 0.00									
age													318	0.03	-0.05, 0.11	0.5				
MoCA_BL																	318	-0.07	-0.22, 0.09	0.4
R^2		0.593				0.722				0.723				0.722				0.723		
Adjusted R^2		0.590				0.718				0.719				0.718				0.718		
AIC		-869				-988				-988				-986				-986		
σ		0.061				0.051				0.051				0.051				0.051		
**Likelihood ratio test**																				
**p-value**						**<0.001**				0.182				0.507				0.395		

Table S1.1.4: Model optimization for predicting 2-back C across gameplays. We fitted different linear models to predict the response bias C in the 2-back task with Gameplay (1–160), Group (Active tACS vs. Sham tACS), age, and baseline Montreal Cognitive Assessment (MoCA_BL) score. The table contains full results of the predictors and model metrics (R^2, adjusted R^2, AIC, sigma σ). Models were compared using the likelihood ratio test (*model.comparison* in R), assessing the significant impact of a specific term added to the model (adapted model) versus the model without this term (current best model). The p-value of the likelihood ratio test refers to the comparison between the new, adapted model with the last best model (from left to right). $p \leq 0.05$ indicates statistical significance (highlighted in bold). For the prediction of 2-back C, this optimization process resulted in the following best model: C ~ Gameplay × Group + Gameplay². tACS = transcranial alternating current stimulation; CI = confidence interval; AIC = Akaike Information Criteria.

S1.1.5 Reaction Time in the 1-back Task Across Gameplays

	RT ~ gameplay × group				...+ gameplay²				...+ gameplay² × group				...+ gameplay² + age				...+ gameplay² + age + MoCA_BL			
**Predictor**	**N**	**Beta**	**95% CI**	**p-value**	**N**	**Beta**	**95% CI**	**p-value**	**N**	**Beta**	**95% CI**	**p-value**	**N**	**Beta**	**95% CI**	**p-value**	**N**	**Beta**	**95% CI**	**p-value**
gameplay	7,123	0.00	0.00, 0.00	**<0.001**	7,123	-0.01	-0.01, -0.01	**<0.001**	7,123	-0.01	-0.01, -0.01	**<0.001**	7,123	-0.01	-0.01, -0.01	**<0.001**	7,123	-0.01	-0.01, -0.01	**<0.001**
group				**<0.001**				**<0.001**				**<0.001**				**<0.001**				**<0.001**
Sham tACS	3,579	—	—		3,579	—	—		3,579	—	—		3,579	—	—		3,579	—	—	
Active tACS	3,544	-0.07	-0.09, -0.04		3,544	-0.07	-0.09, -0.04		3,544	-0.08	-0.12, -0.05		3,544	-0.05	-0.07, -0.03		3,544	-0.06	-0.08, -0.04	
gameplay * group	7,123			**0.018**	7,123			**0.024**	7,123			**0.035**	7,123			0.053	7,123			0.062
gameplay * Active tACS	3,544	0.00	0.00, 0.00		3,544	0.00	0.00, 0.00		3,544	0.00	0.00, 0.00		3,544	0.00	0.00, 0.00		3,544	0.00	0.00, 0.00	
I(gameplay²)					7,123	0.00	0.00, 0.00	**<0.001**	7,123	0.00	0.00, 0.00	**<0.001**	7,123	0.00	0.00, 0.00	**<0.001**	7,123	0.00	0.00, 0.00	**<0.001**
group * I(gameplay²)									7,123			0.11								
Active tACS * I(gameplay²)									3,544	0.00	0.00, 0.00									
age													7,123	0.02	0.02, 0.02	**<0.001**	7,123	0.01	0.01, 0.01	**<0.001**
MoCA_BL																	7,123	-0.03	-0.03, -0.02	**<0.001**
Nagelkerke's R²		0.22				0.24				0.24				0.43				0.5		
AIC		94,487				94,329				94,328				92,269				91,363		
Deviance		387				379				379				284				250		
**Likelihood ratio test**																				
**p-value**						**<0.001**				0.112				**<0.001**				**<0.001**		

Table S1.1.5: Model optimization for predicting 1-back reaction time across gameplays. We fitted different generalized linear models to predict the reaction time (RT) in the 1-back task with Gameplay (1–96), Group (Active tACS vs. Sham tACS), age, and baseline Montreal Cognitive Assessment (MoCA_BL) score. The table contains full results of the predictors and model metrics (Nagelkerke's R^2, AIC, deviance). Models were compared using the likelihood ratio test (*model.comparison* in R), assessing the significant impact of a specific term added to the model (adapted model) versus the model without this term (current best model). The p-value of the likelihood ratio test refers to the comparison between the new, adapted model with the last best model (from left to right). $p \leq 0.05$ indicates statistical significance (highlighted in bold). For the prediction of 1-back RT, this optimization process resulted in the following best model: RT ~ Gameplay × Group + Gameplay^2 + age + $\text{MoCA}_{\text{baseline}}$. tACS = transcranial alternating current stimulation; CI = confidence interval; AIC = Akaike Information Criteria.

S1.1.6 Reaction Time in the 2-back Task Across Gameplays

	RT ~ gameplay × group				...+ gameplay²				...+ gameplay² × group				...+ gameplay² × group + age				...+ gameplay² × group + age + MoCA_BL			
**Predictor**	**N**	**Beta**	**95% CI**	**p-value**	**N**	**Beta**	**95% CI**	**p-value**	**N**	**Beta**	**95% CI**	**p-value**	**N**	**Beta**	**95% CI**	**p-value**	**N**	**Beta**	**95% CI**	**p-value**
gameplay	12,081	0.00	0.00, 0.00	**<0.001**	12,081	-0.01	-0.01, -0.01	**<0.001**	12,081	-0.01	-0.01, -0.01	**<0.001**	12,081	-0.01	-0.01, -0.01	**<0.001**	12,081	-0.01	-0.01, -0.01	**<0.001**
group				0.14				0.2				**0.001**				**0.014**				**0.007**
Sham tACS	6,125	—	—		6,125	—	—		6,125	—	—		6,125	—	—		6,125	—	—	
Active tACS	5,956	-0.02	-0.04, 0.01		5,956	-0.02	-0.04, 0.01		5,956	-0.06	-0.10, -0.03		5,956	-0.05	-0.08, -0.01		5,956	-0.05	-0.08, -0.01	
gameplay * group	12,081			**0.002**	12,081			**0.003**	12,081			**<0.001**	12,081			**<0.001**	12,081			**<0.001**
gameplay * Active tACS	5,956	0.00	0.00, 0.00		5,956	0.00	0.00, 0.00		5,956	0.00	0.00, 0.00		5,956	0.00	0.00, 0.00		5,956	0.00	0.00, 0.00	
I(gameplay²)					12,081	0.00	0.00, 0.00	**<0.001**	12,081	0.00	0.00, 0.00	**<0.001**	12,081	0.00	0.00, 0.00	**<0.001**	12,081	0.00	0.00, 0.00	**<0.001**
group * I(gameplay²)									12,081			**0.001**	12,081			**<0.001**	12,081			**<0.001**
Active tACS * I(gameplay²)									5,956	0.00	0.00, 0.00		5,956	0.00	0.00, 0.00		5,956	0.00	0.00, 0.00	
age													12,081	0.01	0.01, 0.01	**<0.001**	12,081	0.01	0.01, 0.01	**<0.001**
MoCA_BL																	12,081	-0.01	-0.01, -0.01	**<0.001**
Nagelkerke's R²		0.27				0.29				0.29				0.35				0.35		
AIC		188,920				188,543				188,533				187,579				187,512		
Deviance		1,359				1,318				1,316				1,218				1,211		
**Likelihood ratio test**																				
**p-value**						**<0.001**				**0.001**				**<0.001**				**<0.001**		

Table S1.1.6: Model optimization for predicting 2-back reaction time across gameplays. We fitted different generalized linear models to predict the reaction time (RT) in the 1-back task with Gameplay (1–160), Group (Active tACS vs. Sham tACS), age, and baseline Montreal Cognitive Assessment (MoCA_BL) score. The table contains full results of the predictors and model metrics (Nagelkerke's R^2, AIC, deviance). Models were compared using the likelihood ratio test (*model.comparison* in R), assessing the significant impact of a specific term added to the model (adapted model) versus the model without this term (current best model). The p-value of the likelihood ratio test refers to the comparison between the new, adapted model with the last best model (from left to right). $p \leq 0.05$ indicates statistical significance (highlighted in bold). For the prediction of 2-back RT, this optimization process resulted in the following best model: RT ~ Gameplay × Group + Gameplay² × Group + age + $MoCA_{baseline}$. tACS = transcranial alternating current stimulation; CI = confidence interval; AIC = Akaike Information Criteria.

S1.2 Statistical Models Predicting Between-Group n-back Performances at Stimulation Sessions

	d' ~ Stim. session × group				...+ age				...+ age + MoCA_BL			
**Predictor**	**N**	**Beta**	**95% CI**	**p-value**	**N**	**Beta**	**95% CI**	**p-value**	**N**	**Beta**	**95% CI**	**p-value**
stim_session				**<0.001**				**<0.001**				**<0.001**
1	18	—	—		18	—	—		18	—	—	
2	20	0.02	-0.04, 0.08		20	0.03	-0.03, 0.09		20	0.03	-0.03, 0.09	
3	20	0.01	-0.05, 0.07		20	0.02	-0.04, 0.08		20	0.02	-0.04, 0.08	
4	20	0.12	0.06, 0.18		20	0.12	0.06, 0.18		20	0.12	0.06, 0.18	
5	20	0.13	0.07, 0.19		20	0.14	0.08, 0.20		20	0.14	0.08, 0.20	
6	20	0.21	0.15, 0.27		20	0.21	0.15, 0.27		20	0.21	0.15, 0.27	
7	20	0.23	0.17, 0.29		20	0.23	0.17, 0.29		20	0.23	0.17, 0.29	
8	20	0.28	0.22, 0.34		20	0.28	0.22, 0.35		20	0.29	0.23, 0.35	
9	20	0.27	0.21, 0.33		20	0.27	0.21, 0.33		20	0.27	0.21, 0.33	
10	20	0.36	0.30, 0.42		20	0.36	0.30, 0.42		20	0.36	0.30, 0.43	
11	20	0.32	0.26, 0.38		20	0.32	0.26, 0.38		20	0.32	0.26, 0.39	
12	20	0.33	0.27, 0.39		20	0.34	0.28, 0.40		20	0.34	0.28, 0.40	
13	20	0.41	0.35, 0.47		20	0.41	0.35, 0.47		20	0.41	0.35, 0.47	
14	20	0.36	0.30, 0.42		20	0.36	0.30, 0.42		20	0.36	0.30, 0.42	
15	20	0.33	0.27, 0.39		20	0.33	0.27, 0.39		20	0.33	0.27, 0.39	
16	20	0.39	0.33, 0.45		20	0.39	0.33, 0.45		20	0.39	0.33, 0.45	
group				>0.9				0.062				0.11
Active tACS	159	—	—		159	—	—		159	—	—	
Sham tACS	159	0.00	-0.06, 0.06		159	-0.10	-0.21, 0.01		159	-0.13	-0.29, 0.03	
stim_session * group	318			**.015**	318			**.009**	318			**0.008**
2 * Sham tACS	10	-0.09	-0.17, 0.00		10	-0.10	-0.18, -0.01		10	-0.10	-0.19, -0.01	
3 * Sham tACS	10	-0.03	-0.12, 0.06		10	-0.04	-0.13, 0.04		10	-0.04	-0.13, 0.04	
4 * Sham tACS	10	-0.01	-0.09, 0.08		10	-0.02	-0.11, 0.07		10	-0.02	-0.11, 0.07	
5 * Sham tACS	10	-0.03	-0.11, 0.06		10	-0.04	-0.13, 0.04		10	-0.04	-0.13, 0.04	
6 * Sham tACS	10	-0.09	-0.18, 0.00		10	-0.10	-0.19, -0.02		10	-0.10	-0.19, -0.02	
7 * Sham tACS	10	-0.06	-0.14, 0.03		10	-0.07	-0.16, 0.01		10	-0.07	-0.16, 0.01	
8 * Sham tACS	10	-0.08	-0.17, 0.00		10	-0.10	-0.19, -0.01		10	-0.10	-0.19, -0.01	
9 * Sham tACS	10	-0.10	-0.18, -0.01		10	-0.11	-0.20, -0.03		10	-0.11	-0.20, -0.03	
10 * Sham tACS	10	-0.14	-0.23, -0.06		10	-0.16	-0.25, -0.07		10	-0.16	-0.25, -0.08	
11 * Sham tACS	10	-0.07	-0.16, 0.01		10	-0.09	-0.17, 0.00		10	-0.09	-0.18, 0.00	
12 * Sham tACS	10	-0.10	-0.19, -0.01		10	-0.11	-0.20, -0.03		10	-0.11	-0.20, -0.03	
13 * Sham tACS	10	-0.13	-0.22, -0.05		10	-0.15	-0.23, -0.06		10	-0.15	-0.23, -0.06	
14 * Sham tACS	10	-0.06	-0.15, 0.02		10	-0.08	-0.16, 0.01		10	-0.08	-0.17, 0.01	
15 * Sham tACS	10	-0.03	-0.11, 0.06		10	-0.04	-0.13, 0.05		10	-0.04	-0.13, 0.05	
16 * Sham tACS	10	-0.11	-0.19, -0.02		10	-0.11	-0.19, -0.02		10	-0.11	-0.19, -0.02	
age					318	0.13	0.02, 0.24	**0.024**	318	0.15	0.00, 0.31	0.051
MoCA_BL									318	0.07	-0.23, 0.37	0.6
R^2		0.802				0.805				0.805		
Adjusted R^2		0.780				0.783				0.783		
AIC		-783				-787				-785		
σ		0.067				0.067				0.067		
**Likelihood ratio test**												
**p-value**						**0.024**				0.649		

Table S1.2.1: Model optimization for predicting 2-back *d'* at stimulation sessions. We fitted different linear models to predict the sensitivity index *d'* in the 2-back task with Stimulation Session

(1–16), Group (Active tACS vs. Sham tACS), age, and baseline Montreal Cognitive Assessment (MoCA) score. The table contains full results of the predictors and model metrics (R^2, adjusted R^2, AIC, sigma σ). Models were compared using the likelihood ratio test (*model.comparison* in R), assessing the significant impact of a specific term added to the model (adapted model) versus the model without this term (current best model). The p-value of the likelihood ratio test refers to the comparison between the new, adapted model with the last best model (from left to right). $p \leq 0.05$ indicates statistical significance (highlighted in bold). For the prediction of 2-back *d*', this optimization process resulted in the following best model: *d*' ~ Stimulation Session × Group + age. tACS = transcranial alternating current stimulation; CI = confidence interval; AIC = Akaike Information Criteria.

Stimulation session	Effect	EMMeans Difference	lower.CI	upper.CI	p-value	Cohen's d effect size
1	Active tACS - Sham tACS	0.10	-0.01	0.21	0.062	1.50
2	Active tACS - Sham tACS	0.20	0.09	0.31	**0.001**	2.97
3	Active tACS - Sham tACS	0.14	0.03	0.26	**0.013**	2.16
4	Active tACS - Sham tACS	0.12	0.01	0.23	**0.039**	1.81
5	Active tACS - Sham tACS	0.14	0.03	0.26	**0.015**	2.14
6	Active tACS - Sham tACS	0.20	0.09	0.32	< **0.001**	3.04
7	Active tACS - Sham tACS	0.17	0.06	0.29	**0.003**	2.58
8	Active tACS - Sham tACS	0.20	0.08	0.32	**0.001**	3.02
9	Active tACS - Sham tACS	0.21	0.10	0.33	< **0.001**	3.20
10	Active tACS - Sham tACS	0.26	0.14	0.38	< **0.001**	3.92
11	Active tACS - Sham tACS	0.19	0.07	0.30	**0.001**	2.83
12	Active tACS - Sham tACS	0.21	0.10	0.33	< **0.001**	3.19
13	Active tACS - Sham tACS	0.25	0.13	0.36	< **0.001**	3.70
14	Active tACS - Sham tACS	0.18	0.06	0.29	**0.002**	2.67
15	Active tACS - Sham tACS	0.14	0.03	0.26	**0.016**	2.11
16	Active tACS - Sham tACS	0.21	0.10	0.31	< **0.001**	3.10

Table S1.2.2: Post hoc pairwise comparisons based on the optimized model predicting 2-back *d*' at stimulation sessions. Based on the optimized model to predict the sensitivity index *d*' in the 2-back task (see table S1.2.1), we calculated estimated marginal means (EMMs) and performed post hoc pairwise comparisons between the active tACS and the sham tACS group at each stimulation session. The p-values have been Bonferroni-corrected. $p \leq 0.05$ indicates statistical

significance (highlighted in bold). Effect sizes were computed utilizing Cohen's d. tACS = transcranial alternating current stimulation; CI = confidence interval.

	C ~ Stim. session × group				...+ age				...+ MoCA_BL			
**Predictor**	**N**	**Beta**	**95% CI**	**p-value**	**N**	**Beta**	**95% CI**	**p-value**	**N**	**Beta**	**95% CI**	**p-value**
stim_session				**<0.001**				**<0.001**				**<0.001**
1	18	—	—		18	—	—		18	—	—	
2	20	0.02	-0.02, 0.07		20	0.02	-0.02, 0.07		20	0.02	-0.02, 0.07	
3	20	-0.09	-0.14, -0.05		20	-0.09	-0.14, -0.05		20	-0.09	-0.14, -0.05	
4	20	-0.18	-0.22, -0.13		20	-0.18	-0.22, -0.13		20	-0.18	-0.22, -0.13	
5	20	-0.17	-0.22, -0.13		20	-0.17	-0.21, -0.12		20	-0.17	-0.22, -0.13	
6	20	-0.18	-0.23, -0.14		20	-0.18	-0.23, -0.14		20	-0.18	-0.23, -0.14	
7	20	-0.19	-0.23, -0.14		20	-0.18	-0.23, -0.14		20	-0.19	-0.23, -0.14	
8	20	-0.22	-0.26, -0.17		20	-0.22	-0.26, -0.17		20	-0.22	-0.26, -0.17	
9	20	-0.23	-0.27, -0.18		20	-0.23	-0.27, -0.18		20	-0.23	-0.28, -0.18	
10	20	-0.24	-0.28, -0.19		20	-0.23	-0.28, -0.19		20	-0.24	-0.28, -0.19	
11	20	-0.26	-0.30, -0.21		20	-0.26	-0.30, -0.21		20	-0.26	-0.31, -0.22	
12	20	-0.28	-0.32, -0.23		20	-0.28	-0.32, -0.23		20	-0.28	-0.33, -0.24	
13	20	-0.28	-0.32, -0.23		20	-0.28	-0.32, -0.23		20	-0.28	-0.33, -0.24	
14	20	-0.28	-0.33, -0.24		20	-0.28	-0.32, -0.23		20	-0.28	-0.33, -0.24	
15	20	-0.29	-0.33, -0.24		20	-0.29	-0.33, -0.24		20	-0.29	-0.33, -0.24	
16	20	-0.31	-0.35, -0.26		20	-0.31	-0.35, -0.26		20	-0.31	-0.35, -0.26	
group				0.064				>0.9				**0.041**
Active tACS	159	—	—		159	—	—		159	—	—	
Sham tACS	159	0.04	0.00, 0.09		159	0.00	-0.08, 0.08		159	0.05	0.00, 0.10	
stim_session * group	318			**<0.001**	318			**<0.001**	318			**<0.001**
2 * Sham tACS	10	-0.13	-0.20, -0.07		10	-0.14	-0.20, -0.07		10	-0.13	-0.19, -0.07	
3 * Sham tACS	10	-0.05	-0.12, 0.01		10	-0.06	-0.12, 0.01		10	-0.06	-0.12, 0.01	
4 * Sham tACS	10	0.00	-0.06, 0.06		10	-0.01	-0.07, 0.06		10	0.00	-0.07, 0.06	
5 * Sham tACS	10	-0.01	-0.08, 0.05		10	-0.02	-0.08, 0.05		10	-0.01	-0.08, 0.05	
6 * Sham tACS	10	-0.03	-0.09, 0.04		10	-0.03	-0.09, 0.03		10	-0.03	-0.09, 0.04	
7 * Sham tACS	10	-0.04	-0.11, 0.02		10	-0.05	-0.11, 0.01		10	-0.05	-0.11, 0.02	
8 * Sham tACS	10	-0.04	-0.10, 0.03		10	-0.04	-0.11, 0.02		10	-0.04	-0.10, 0.02	
9 * Sham tACS	10	0.00	-0.07, 0.06		10	-0.01	-0.07, 0.06		10	0.00	-0.07, 0.06	
10 * Sham tACS	10	0.01	-0.05, 0.07		10	0.00	-0.06, 0.07		10	0.01	-0.06, 0.07	
11 * Sham tACS	10	0.02	-0.04, 0.08		10	0.01	-0.05, 0.08		10	0.02	-0.05, 0.08	
12 * Sham tACS	10	0.03	-0.04, 0.09		10	0.02	-0.04, 0.08		10	0.02	-0.04, 0.09	
13 * Sham tACS	10	0.04	-0.03, 0.10		10	0.03	-0.03, 0.10		10	0.04	-0.03, 0.10	
14 * Sham tACS	10	0.04	-0.02, 0.10		10	0.03	-0.03, 0.10		10	0.04	-0.03, 0.10	
15 * Sham tACS	10	0.08	0.02, 0.14		10	0.07	0.01, 0.14		10	0.08	0.01, 0.14	
16 * Sham tACS	10	0.08	0.01, 0.14		10	0.08	0.02, 0.14		10	0.08	0.02, 0.14	
age					318	0.05	-0.03, 0.13	0.2				
MoCA_BL									318	-0.07	-0.23, 0.09	0.4
R^2		0.755				0.756				0.755		
Adjusted R^2		0.728				0.729				0.728		
AIC		-974				-973				-973		
σ		0.050				0.050				0.050		
**Likelihood ratio test p-value**						0.216				0.387		

Table S1.2.3: Model optimization for predicting 2-back C at stimulation sessions. We fitted different linear models to predict the response bias C in the 2-back task with Stimulation Session (1–16), Group (Active tACS vs. Sham tACS), age, and baseline Montreal Cognitive Assessment

(MoCA) score. The table contains full results of the predictors and model metrics (R^2, adjusted R^2, AIC, sigma σ). Models were compared using the likelihood ratio test (*model.comparison* in R), assessing the significant impact of a specific term added to the model (adapted model) versus the model without this term (current best model). The p-value of the likelihood ratio test refers to the comparison between the new, adapted model with the last best model (from left to right). $p \leq 0.05$ indicates statistical significance (highlighted in bold). For the prediction of 2-back C, this optimization process resulted in the following best model: C ~ Stimulation Session × Group. tACS = transcranial alternating current stimulation; CI = confidence interval; AIC = Akaike Information Criteria.

Stimulation session	Effect	EMMeans Difference	lower.CI	upper.CI	p-value	Cohen's d effect size
1	Active tACS - Sham tACS	-0.04	-0.09	0.00	0.064	-0.88
2	Active tACS - Sham tACS	0.09	0.04	0.13	< **0.001**	1.77
3	Active tACS - Sham tACS	0.01	-0.03	0.05	0.653	0.20
4	Active tACS - Sham tACS	-0.04	-0.09	0.00	0.054	-0.87
5	Active tACS - Sham tACS	-0.03	-0.08	0.01	0.152	-0.64
6	Active tACS - Sham tACS	-0.02	-0.06	0.03	0.409	-0.37
7	Active tACS - Sham tACS	0.00	-0.04	0.04	0.983	0.01
8	Active tACS - Sham tACS	-0.01	-0.05	0.04	0.753	-0.14
9	Active tACS - Sham tACS	-0.04	-0.09	0.00	0.062	-0.84
10	Active tACS - Sham tACS	-0.05	-0.10	-0.01	**0.016**	-1.08
11	Active tACS - Sham tACS	-0.06	-0.11	-0.02	**0.005**	-1.27
12	Active tACS - Sham tACS	-0.07	-0.11	-0.03	**0.002**	-1.39
13	Active tACS - Sham tACS	-0.08	-0.12	-0.04	< **0.001**	-1.63
14	Active tACS - Sham tACS	-0.08	-0.13	-0.04	< **0.001**	-1.70
15	Active tACS - Sham tACS	-0.12	-0.17	-0.08	< **0.001**	-2.46
16	Active tACS - Sham tACS	-0.12	-0.17	-0.08	< **0.001**	-2.45

Table S1.2.4: Post hoc pairwise comparisons based on the optimized model predicting 2-back C at stimulation sessions. Based on the optimized model to predict the response bias C in the 2-back task (see table S1.2.3), we calculated estimated marginal means (EMMs) and performed post hoc pairwise comparisons between the Active tACS and the Sham tACS group at each stimulation session. The p-values have been Bonferroni-corrected. $p \leq 0.05$ indicates statistical significance

(highlighted in bold). Effect sizes were computed utilizing Cohen's d. tACS = transcranial alternating current stimulation; CI = confidence interval.

	**RT ~ Stim. session × group**				**...+ age**				**...+ age + MoCA_BL**			
**Predictor**	**N**	**Beta**	**95% CI**	**p-value**	**N**	**Beta**	**95% CI**	**p-value**	**N**	**Beta**	**95% CI**	**p-value**
stim_session				**<0.001**				**<0.001**				**<0.001**
1	637	—	—		637	—	—		637	—	—	
2	728	-0.13	-0.19, -0.08		728	-0.13	-0.18, -0.08		728	-0.13	-0.18, -0.08	
3	752	-0.23	-0.29, -0.18		752	-0.24	-0.29, -0.18		752	-0.24	-0.29, -0.19	
4	761	-0.27	-0.32, -0.21		761	-0.27	-0.32, -0.22		761	-0.27	-0.32, -0.22	
5	764	-0.34	-0.40, -0.29		764	-0.35	-0.40, -0.30		764	-0.35	-0.40, -0.30	
6	767	-0.39	-0.44, -0.34		767	-0.40	-0.45, -0.35		767	-0.40	-0.45, -0.35	
7	769	-0.43	-0.48, -0.38		769	-0.44	-0.49, -0.39		769	-0.44	-0.49, -0.39	
8	768	-0.46	-0.51, -0.41		768	-0.47	-0.52, -0.42		768	-0.47	-0.52, -0.42	
9	767	-0.50	-0.55, -0.45		767	-0.51	-0.56, -0.46		767	-0.51	-0.56, -0.46	
10	768	-0.52	-0.58, -0.47		768	-0.53	-0.59, -0.48		768	-0.54	-0.59, -0.49	
11	770	-0.57	-0.62, -0.52		770	-0.58	-0.63, -0.53		770	-0.58	-0.63, -0.53	
12	768	-0.58	-0.63, -0.52		768	-0.59	-0.64, -0.54		768	-0.59	-0.64, -0.54	
13	769	-0.61	-0.66, -0.56		769	-0.62	-0.67, -0.57		769	-0.63	-0.68, -0.58	
14	770	-0.64	-0.69, -0.58		770	-0.65	-0.70, -0.60		770	-0.65	-0.70, -0.60	
15	769	-0.64	-0.69, -0.59		769	-0.65	-0.70, -0.60		769	-0.66	-0.71, -0.61	
16	754	-0.66	-0.72, -0.61		754	-0.68	-0.73, -0.63		754	-0.68	-0.73, -0.63	
group				0.073				0.2				0.2
Active tACS	5,956	—	—		5,956	—	—		5,956	—	—	
Sham tACS	6,125	0.05	0.00, 0.11		6,125	0.03	-0.02, 0.09		6,125	0.04	-0.02, 0.09	
stim_session * group	12,081			0.068	12,081			0.084	12,081			0.066
2 * Sham tACS	369	-0.02	-0.10, 0.05		369	-0.02	-0.10, 0.05		369	-0.02	-0.09, 0.05	
3 * Sham tACS	380	-0.01	-0.09, 0.06		380	-0.01	-0.08, 0.06		380	-0.01	-0.08, 0.06	
4 * Sham tACS	386	-0.03	-0.11, 0.04		386	-0.03	-0.10, 0.04		386	-0.03	-0.10, 0.04	
5 * Sham tACS	389	-0.06	-0.13, 0.02		389	-0.06	-0.13, 0.02		389	-0.06	-0.13, 0.01	
6 * Sham tACS	388	-0.10	-0.18, -0.03		388	-0.09	-0.17, -0.02		388	-0.09	-0.16, -0.02	
7 * Sham tACS	390	-0.08	-0.15, 0.00		390	-0.07	-0.14, 0.00		390	-0.07	-0.14, 0.00	
8 * Sham tACS	390	-0.09	-0.16, -0.01		390	-0.08	-0.16, -0.01		390	-0.08	-0.15, -0.01	
9 * Sham tACS	388	-0.09	-0.17, -0.02		388	-0.09	-0.16, -0.02		388	-0.09	-0.16, -0.02	
10 * Sham tACS	390	-0.09	-0.16, -0.01		390	-0.08	-0.15, -0.01		390	-0.08	-0.15, -0.01	
11 * Sham tACS	390	-0.09	-0.16, -0.01		390	-0.08	-0.15, -0.01		390	-0.08	-0.15, -0.01	
12 * Sham tACS	389	-0.12	-0.19, -0.04		389	-0.11	-0.18, -0.04		389	-0.11	-0.18, -0.04	
13 * Sham tACS	390	-0.06	-0.14, 0.01		390	-0.05	-0.13, 0.02		390	-0.05	-0.12, 0.02	
14 * Sham tACS	390	-0.05	-0.13, 0.02		390	-0.04	-0.12, 0.03		390	-0.04	-0.11, 0.03	
15 * Sham tACS	389	-0.08	-0.16, -0.01		389	-0.07	-0.14, 0.00		389	-0.07	-0.14, 0.00	
16 * Sham tACS	381	-0.07	-0.14, 0.01		381	-0.06	-0.13, 0.01		381	-0.06	-0.13, 0.01	
age					12,081	0.01	0.01, 0.01	**<0.001**	12,081	0.01	0.01, 0.01	**<0.001**
MoCA_BL									12,081	-0.01	-0.01, -0.01	**<0.001**
Nagelkerke's R^2		0.29				0.34				0.35		
AIC		188,607				187,659				187,593		
Deviance		1,319				1,221				1,214		
**Likelihood ratio test**												
**p-value**						**<0.001**				**<0.001**		

Table S1.2.5: Model optimization for predicting 2-back reaction time at stimulation sessions. We fitted different generalized linear models to predict the reaction time (RT) in the 2-back task with Stimulation Session (1–16), Group (Active tACS vs. Sham tACS), age, and baseline Montreal Cognitive Assessment (MoCA) score. The table contains full results of the predictors and model

metrics (Nagelkerke's R^2, AIC, deviance). Models were compared using the likelihood ratio test (*model.comparison* in R), assessing the significant impact of a specific term added to the model (adapted model) versus the model without this term (current best model). The p-value of the likelihood ratio test refers to the comparison between the new, adapted model with the last best model (from left to right). $p \leq 0.05$ indicates statistical significance (highlighted in bold). For the prediction of 2-back RT, this optimization process resulted in the following best model: RT ~ Stimulation Session × Group + age + $MoCA_{baseline}$. tACS = transcranial alternating current stimulation; CI = confidence interval; AIC = Akaike Information Criteria.

Stimulation session	Effect	EMMeans Difference	lower.CI	upper.CI	p-value	Cohen's d effect size
1	Active tACS - Sham tACS	-0.04	-0.09	0.02	0.18	-0.11
2	Active tACS - Sham tACS	-0.01	-0.06	0.04	0.58	-0.04
3	Active tACS - Sham tACS	-0.03	-0.07	0.02	0.28	-0.08
4	Active tACS - Sham tACS	-0.01	-0.05	0.04	0.85	-0.01
5	Active tACS - Sham tACS	0.02	-0.03	0.07	0.41	0.06
6	Active tACS - Sham tACS	0.06	0.01	0.10	**0.02**	0.18
7	Active tACS - Sham tACS	0.04	-0.01	0.08	0.14	0.11
8	Active tACS - Sham tACS	0.05	0.00	0.09	**0.049**	0.15
9	Active tACS - Sham tACS	0.05	0.01	0.10	**0.03**	0.17
10	Active tACS - Sham tACS	0.05	-0.00	0.09	0.06	0.14
11	Active tACS - Sham tACS	0.04	-0.00	0.09	0.07	0.14
12	Active tACS - Sham tACS	0.07	0.03	0.12	**0.002**	0.23
13	Active tACS - Sham tACS	0.02	-0.03	0.07	0.45	0.06
14	Active tACS - Sham tACS	0.01	-0.04	0.06	0.71	0.03
15	Active tACS - Sham tACS	0.04	-0.01	0.08	0.12	0.12
16	Active tACS - Sham tACS	0.02	-0.03	0.07	0.36	0.07

Table S1.2.6: Post hoc pairwise comparisons based on the optimized model predicting 2-back reaction time at stimulation sessions. Based on the optimized model to predict the reaction time (RT) in the 2-back task (see table S1.2.5), we calculated estimated marginal means (EMMs) and performed post hoc pairwise comparisons between the Active tACS and the Sham tACS group at each stimulation session. The p-values have been Bonferroni-corrected. $p \leq 0.05$ indicates statistical significance (highlighted in bold). Effect sizes were computed utilizing Cohen's d. tACS = transcranial alternating current stimulation; CI = confidence interval.

S2. Side Effects and Adverse Effects

	Active tACS (n = 38)	Sham tACS (n = 39)	p-value
**Side effects**			
Phosphenes	6 (1%)	9 (1%)	0.69
Skin irritation	3 (<1%)	10 (2%)	0.22
Tingling	377 (62%)	404 (65%)	0.72
**Adverse effects**			
Headache	5 (<1%)	3 (<1%)	0.50
Nervousness	3 (<1%)	0	0.32
Tiredness	8 (1%)	14 (2%)	0.35
Vertigo	2 (<1%)	0	0.16

Table S2: Detailed list of reported side effects and adverse effects as well as comparisons between intervention groups. The data comprises the total counts and corresponding percentages (in parentheses) of reported side effects and adverse effects in both the Active and Sham transcranial alternating current stimulation (tACS) groups. Statistical comparisons between groups were conducted using unpaired two-sided t-tests, with a significance threshold set at a p-value of ≤ 0.05.

References

1. Sjoberg DD, Whiting K, Curry M, Lavery JA, Larmarange J. Reproducible summary tables with the gtsummary package. The R Journal 2021;13:570–80. https://doi.org/10.32614/RJ-2021-053.

2. Fife D (2022) Flexplot: Graphically-based data analysis. Psychol Methods 27:477–496. https://doi.org/10.1037/met0000424
